# Effect of pretreatment on bioactive compounds in wild rocket juice

**DOI:** 10.1007/s13197-019-03992-3

**Published:** 2019-08-06

**Authors:** Elżbieta Radziejewska-Kubzdela, Anna Olejnik, Róża Biegańska-Marecik

**Affiliations:** 1 Institute of Technology of Food of Plant Origin, Poznań University of Life Sciences, Wojska Polskiego 31, 60-624 Poznań, Poland; 2 Department of Biotechnology and Food Microbiology, Poznań University of Life Sciences, Wojska Polskiego 48, 60-627 Poznań, Poland

**Keywords:** Wild rocket, Polyphenols, Glucosinolates, Antioxidant capacity

## Abstract

The aim of the study was to determine the effect of pretreatment with hot water or steaming on glucosinolates, polyphenols contents and antioxidant capacity in obtained raw juices. Moreover, in vitro cytotoxic activity of the raw juice to the cells derived from the gastrointestinal tract, including the small intestine (IEC-6 cell line), colon (Caco-2 cell line) and the liver (HepG2 cell line) were also investigated. The dominant glucosinolates in the wild rocket leaves were glucoraphanin (36%) and dimeric 4-mercaptobutyl (30%), followed by glucosativin and glucoerucin, 11% per each. Glucothiobeinin (6%), glucobrassicin (1%), 4-methoxyglucobrassicin (1%) and two unidentified compounds (4%) were also detected in rocket leaves. In terms of phenolic compounds, quercetin constituted the majority (55%) and the rest composed of hydroxycinnamic acids. In raw juices produced from steamed, pretreatment with hot water and untreated (control) leaves, glucosinolate contents were lower about 21%, 37% and 53%, respectively, than their levels in the raw material. The highest content of polyphenols among the juices tested (45.4 mg/100 g fresh weight) and antioxidant capacity (5.8 µmol Trolox/1 g f.w.) was recorded in the raw juice from pretreated leaves with hot water. The wild rocket raw juice concentrations responsible for a 50% reduction in Caco-2 and HepG2 cell viability were estimated at 1.87 ± 0.08 mg/mL and 3.54 ± 0.29 mg/mL. The viability of the IEC-6 cells was reduced by only 19.04%, at the maximum concentration (3.6 mg/mL) of the raw juice.

## Introduction

Wild rocket (*Diplotaxis tenuifolia* (L.) DC.) belongs to the family Brassicaceae. Rocket leaves contain many bioactive compounds, such as polyphenols, especially flavonoids. They protect the colonic epithelium from oxidative damage and contribute to lowering the risk of cardiovascular diseases (Chun et al. 2017). The pungent taste of leaves is linked with the presence of glucosinolates (GLS). The dominant compound is dimeric 4-mercaptobutyl GLS (glucosativin), probably derived from the S-demethylation of 4-methylthiobutyl GLS (glucoerucin) (Bennett et al. 2006). GLS are located in plant vacuoles. A disruption of tissue continuity (e.g. comminution) facilitates their hydrolysis by myrosinase enzyme present in the cytosol or myrosin cells (Radziejewska–Kubzdela and Olejnik 2016). Breakdown products of these compounds have potentially beneficial health effects associated with their anti-cancer activity. Numerous studies indicate a correlation between the potential to lower the risk of certain cancers (e.g. breast, cervical, prostate, lung, stomach or colon) and increased consumption of Brassicaceae vegetables, constituting their good sources (van Poppel et al. 1999; London et al. 2000; Giovannucci et al. 2003; Sapone et al. 2007). Degradation products of GLS can inhibit carcinogenesis and different development stages of the illness.

Rocket is consumed in many countries in the minimally processed form. An interesting alternative seems to be connected with the use of this raw material to produce juice, which may be a part of vegetable or fruit juices. The juice yield may be increased by heat pretreatment of leaves, as indicated by such an effect observed in the case of carrot or red beetroot mash (Zadernowski and Oszmiański 1994; Mannozzi et al. 2018). The increase of yield often leads to improve juice quality with regard to the content of bioactive compounds. In the literature there is no data on either the content or profile of phenolic compounds, GLS in wild rocket juice, which is used in green vegetable juices.

The aim of the study was to determine the effect of pretreatment with hot water or steaming of leaves on the contents of phenolic compounds and GLS as well as antioxidant capacity in the produced raw juices. The tested parameters were determined in the raw material, in the mashes obtained after the comminution of the pretreated leaves and in raw juices. Currently used plants in new forms may induce cytotoxic effects on both normal and cancer cells, especially on cells of the digestive system. Therefore, the cytotoxicity of wild-rocket juice to small intestine cells (IEC-6 cell line), hepatocellular carcinoma cells (HepG2 cell line) and colon cancer cells (Caco-2 cell line) was investigated in this work.

## Materials and methods

### Raw material

Wild rocket leaves (*Diplotaxis tenuifolia* (L.) DC.) were purchased from a horticultural farm located near Poznań, Poland. Leaves with defects were removed manually.

### Chemicals

HPLC gradient grade acetonitrile, ≥ 99.9% (CH_3_CN) and methanol (MeOH) were purchased from Sigma Aldrich ChemieGmbH (Steinheim, Germany). 2,2-azinobis-(3-ethylbenzothiazoline-6-sulphonic acid) (ABTS), 6-hydroxy-2,5,7,8-tetramethylchroman-2-carboxylic acid (Trolox), HPLC grade acetic acid (CH_2_O_2_), DEAE Sephadex A-25, purified sulphatase from *Helix pomatia* and standards for phenolic compounds were supplied from Sigma Aldrich Chemie Co. (St. Louis, USA). Chlorogenic acid was obtained from Sigma Aldrich Chemie Co. (Buchs, Switzerland). Monobasicpotassium phosphate (KH_2_PO_4_) was provided by Sigma AldrichChemie Co. (Tokyo, Japonia), while sodium acetate (C_2_H_3_NaO_2_) was obtained from Chempur (Piekary Śląskie, Poland), glucotropaeolin from Roth (Karlsruhe, Germany), imidazole (C_3_H_4_N_2_) from Merck Schuchardt OHG (Hohenbrunn, Germany). Ultra pure water was produced in the laboratory using a Direct-Q UV3Water Purification System (Millipore, Billerica, USA).

### Technological process

Wild rocket leaves were washed and cut into pieces of 3 cm in length by hand, then divided into three batches. One bath was put aside without any pretreatment (control). One batch (leaf: water ratio as 90:10, w/w%) was put in hot water (90 °C) for 5 min. The last third batch was steamed for 10 min in a Thermomix TM6 (Wuppertal, Vorwerk, Germany). Then, leaves from all batches were comminuted in a Thermomix TM6 for 1 min and each batch was divided into two parts. One part was cooled in tap water, then was frozen in liquid N_2_ and stored at − 50 °C for further analysis. The other part was pressed in a Para-press laboratory press (Arauner Kitzingen, Kitzingein, Germany) at 0.28 MPa for 10 min. The obtained raw juices were frozen in liquid N_2_ and stored at − 50 °C.

### Juice yield

The yield of wild rocket juice (Y) was calculated according to the following equation:

$$ {\text{Yield}}(\% ) = \frac{{{\text{Amount}}\;{\text{of}}\;{\text{juice}}\;{\text{recovered}} }}{{{\text{Amount}}\;{\text{of}}\;{\text{mash}}\;{\text{taken}}}} \times 100$$

(Turk et al. 2010)

### Glucosinolate analysis

All samples were analyzed according to ISO 9167-1 method (ISO9167-1 1992). The analysed samples were freeze-dried (raw material, mashes and raw juices). Next samples (0.5 g) were extracted twice with 3 mL boiling methanol solution (700 g/L) in a water bath at 75 °C for 20 min. Obtained extracts were purified in an ion exchange mini-column (DEAE Sephadex A-25). The column was washed once with 2 mL imidazol formate (6 mol/L) and twice with 1 mL Millipore water and then loaded with 6 mL of each extract. Purified sulphatase from *H. pomatia* was added to the column and incubated overnight at room temperature. A known amount of glucotropaeolin was added to each sample during extraction as the internal standard. Desulphoglucosinolates were eluted with water and were separated using the Agilent Technologies 1200 Rapid Resolution LS system (Waldbronn, Germany) equipped with a Poroshell 120, SB-C18 column (4.6 × 150 mm, 2.7 μm) (Wilmington, USA) at a flow rate of 1 mL/min at 30 °C. The used mobile phase was ultra pure water (A), acetonitrile-ultra pure water mixture (20:80 v/v, B). During 30 min of total run time; the gradient flow was as follows: 100% A/0% B for 1 min, then within 22 min—100% B and within 5 min—100% B, after which the column was equilibrated at 100% A for 2 min. A UV detector was used at a wavelength of 229 nm. Desulphoglucosinolates were quantified with respect to glucotropaeolin and the response factor (ISO9167-1 1992).

### Determination of phenolic compounds

10 g of all samples were weighed and homogenised with 50 mL of 700 g/L methanol using an IKA T-25 homogeniser (Staufen, Germany). Homogenates were shaken for 15 min in a Water Bath Shaker type 357 (Elpin, Lubawa, Poland) and then centrifuged in a MPW-351R centrifuge (Warszawa, Poland) at 3000×*g* for 20 min. Each extraction procedure was performed twice and obtained phenolic extracts were combined then evaporated in a vacuum Büchi R-205 evaporator (Flawil, Switzerland) at 40 °C. Concentrated extracts were diluted up to 25 mL with ultra pure water (Vallejo et al. 2002).

Phenolic compounds were determined by the LC Agilent Technologies 1200 Rapid Resolution system equipped with a Poroshell 120, SB-C18 column (4.6 × 150 mm, 2.7 μm). The mobile phase consisted of 60 g/L acetic acid in 0.002 mol/L sodium acetate (solvent A) and acetonitrile (solvent B) (Tsao and Yang 2003). The flow rate was 1 mL/min and the total run time was 35 min. The system was operated with a gradient flow program: 0–15% B for 15 min, 15–30% B for 25 min, 30–50% B for 5 min and 50–100% B for 5 min. Two phenolic groups (hydroxy-cinnamic acid derivatives at 320 nm as chlorogenic acid equivalents and flavonols at 360 nm as quercetin equivalents) were determined. Total phenolic compounds was calculated as the sum of hydroxycinnamic acid derivatives and flavonols (Tsao and Yang 2003).

### Antioxidant capacity by ABTS^+^: free radical scavenging assay

2.2-Azino-bis-(3-ethylbenzothiazoline-6-sulphonic acid) radical monocation scavenging activity was determined according to a procedure described by Re et al. (1999). ABTS^·+^ was obtained by reacting 0.007 mol/L ABTS water solution with 0.00245 mol/L potassium persulphate. The phenolic extract (50 μL) was mixed with 5 mL diluted ABTS^·+^ solution and its absorption was measured at 734 nm after 6 min at 30 °C. The percentage inhibition of ABTS^·+^ by the phenolic extract was calculated according to the formula:

$$ \%   {\text{Inhibition}} = \left[ {\frac{{Ac_{(0)} - Aa_{(t)} }}{{Ac_{(0)} }}} \right] \times 100 $$

were *Ac*_(0)_ is the absorbance ABTS^·+^ without sample (control) at *t *= 0 min, *Aa*_(t)_ is the absorbance of the phenolic extract at *t *= 6 min. The antioxidant capacity of tested samples, calculated as percentage inhibition of ABTS^·+^, was equated against a Trolox standard curve (1.5–15 µmol/L) (Katalinic et al. 2005). 6-hydroxy-2,5,7,8-tetramethylchroman-2-carboxylic acid (Trolox) was used as a standard and the antioxidant capacity was expressed as micromoles of Trolox per 1 g f.w. (fresh weight).

### Cytotoxic activity of wild rocket raw juice

The Caco-2 (ECACC 86010202), HepG2 (ECACC 85011430) and IEC-6 (ECACC 88071401) cell lines were obtained from the European Collection of Cell Cultures (ECACC) with Sigma–Aldrich (St. Louis, USA) supply. Cells were cultured in Dulbecco’s Modified Eagle’s Medium (DMEM; Sigma–Aldrich), supplemented with 10% heat-inactivated fetal bovine serum (FBS; Gibco, Grand Island, NY, USA), 1% non-essential amino acids 100X (Sigma–Aldrich) and 50 mg/L gentamycin (Gibco, Grand Island, NY, USA). The medium used in the IEC-6 cell culture was also supplemented with 1 μM insulin (Sigma–Aldrich). The cells were maintained at 37 °C in a humidified atmosphere (5% CO_2_, 95% air). The cells were cultured according to the recommendations of ECACC.

The cells were grown in 96-well plates at an initial density of 2.5 × 10^4^ cells/cm^2^. The 24-hour cultures were treated with wild rocket raw juice at concentrations ranging from 0.03 to 3.60 mg/mL and incubated for 48 h under standard culture conditions.

The cytotoxic effect of the raw juice on the viability of Caco-2 and IEC-6 and HepG2 cells was evaluated using an assay based on the cleavage of the yellow dye MTT (3-(4,5-dimethylthiazol-2-yl)-2,5-diphenyltetrazolium bromide; Sigma–Aldrich) to purple formazan crystals by mitochondrial dehydrogenase enzymes, which are active only in living cells (Mosmann 1983). After treatment of the cells with wild rocket raw juice, the MTT solution was added at a concentration 0.5 mg/mL. The cell cultures were incubated at 37 °C for 3 h and then formazan crystals were extracted with acidic isopropanol for 20 min at room temperature. Absorbance was determined at two different wavelengths (570 nm and 690 nm-reference) using a Tecan M200 Infinite microplate reader (Tecan Group Ltd., Männedorf, Switzerland).

Based on the obtained results, dose–response curves were plotted and cytotoxic doses were calculated using the following equations:

$$ \begin{aligned} &{\text{Y}}  = {\text{Y}}_{\hbox{min} } + ({\text{Y}}_{\hbox{max} } - {\text{Y}}_{\hbox{min} } ) /(1 + 10^{{(({\text{LogEC}}_{50} - {\text{LogX}}) \times {\text{s}})}} ), \\ &{\text{LogEC}}_{10}  = {\text{LogEC}}_{50} - (1 / {\text{s}}) \, \times {\text{Log}}(10 /(100 - 10)),\;\;{\text{EC}}_{10} = 10^{{{\text{LogEC}}_{10} }} \\ \end{aligned} $$

where Y_min_ and Y_max_ are the estimated value of the minimal and maximal response, respectively; X is the wild rocket juice concentration; EC_50_ and EC_10_ are the concentrations, at which wild rocket juice reduces cell proliferation and viability by 50% and 10%, respectively; s is the slope of the curve.

### Statistical analysis

The analyses were carried out in triplicate. The analysis of variance (ANOVA) was conducted to determine the significance of the main effects. Differences between the mean values of multiple groups were analysed by parametric Tukey’s post hoc test. Statistical significance was considered at *P* < 0.05. The STATISTICA version 13.1 software (Statsoft, Inc., Tulsa, OK, USA) was used.

## Results and discussion

### Juice yield

Steaming of comminuted leaves resulted in an increase 25% in the raw juice yield when compared to juice yield of untreated leaves (control) (44%). This ratio was 17% in hot water treated samples.

### Glucosinolates profile

The total glucosinolate concentration of raw material was 210 mg/100 g f.w. (Table 1). The recorded glucosinolate content in literature for *Diplotaxis* ranges from 37.4 mg/100 g f.w. to 229 mg/100 g f.w. [calculated on the basis of the dry matter content (8.5%) determined in the tested rocket leaves (data not published)] (Bell et al. 2015; Pasini et al. 2012; Taranto et al. 2016). The percentage contribution of individual glucosinolates to total content was 36% of glucoraphanin, 30% of dimeric 4-mercaptobutyl (DMB-GLS,) 11% of glucosativin, 11% of glucoerucin, 6% of glucothiobeinin, 1% of glucobrassicin and 1% of 4-methoxyglucobrassicin each and 4% of two unidentified compounds (calculated on the basis of values in Table 1). Pasini et al. (2012) in their studies on 37 rocket accessions indicated glucoraphanin (17.6–63%), DMB-GLS (12–49.5%) and glucoerucin (7.7–28.1%,) as dominant compounds. They also detected of glucothiobeinin, 4-OH-glucobrassicin and glucobrassicin. Additionally, they identified glucoalyssin, glucosinalbin, progroitrin and epiprogoitryn, which were not found in the samples tested in this study. Bell et al. (2015) reported glucosativin and DMB-GLS as dominant compounds, (approx. 90% of total glucosinolate content). In study by Taranto et al. (2016), content of glucosativin and DMB-GLS was 51% of total GLS content. Considerable percentage differences for each glucosinolate type in the total content may result from the effect of growing conditions and climatic factors. For instance, Selma et al. (2010) reported the effect of soil amendments prepared from organic wastes (sewage sludge and urban solid waste) on a reduction of individual glucosinolate content in rocket leaves (*Eruca sativa*). Kim et al. (2002) found that glucosinolate content from the edible parts of *Brassica rapa* was strongly affected by N and S applications. Ciska et al. (2000) described the impact of climatic conditions (temperature and precipitation) on the content of GLS in vegetables.

**Table 1:** Glucosinolate profile (mg/100 g f.w.) in mash and obtained juices from wild rocket salad (n = 9, mean ± standard deviation)

Sample	A	B	C	D	E	F	G	H	I	J	Total
raw material	75b ± 2	12.1a ± 0.1	23.9b ± 1.4	22.8ab ± 1.5	5.2c ± 0.2	1.29ab ± 0.03	63a ± 4	1.23ab ± 0.04	nd	5.2c ± 0.3	210d ± 7
M (control)	73b ± 5	10.0b ± 1.1	22.9b ± 3.2	22.9b ± 0.1	1.3ab ± 0.1	0.87a ± 0.07	60a ± 2	1.06a ± 0.12	nd	7.2d ± 0.5	199cd ± 13
MHW	64a ± 2	5.8c ± 0.6	9.9d ± 0.9	24.0abc ± 1.7	1.2a ± 0.3	2.20c ± 0.10	75d ± 1	1.91e ± 0.17	nd	3.8b ± 0.7	188bc ± 7
MSB	49e ± 4	1.5d ± 0.2	5.6a ± 0.7	16.0cd ± 3.0	1.0d ± 0.2	1.27ab ± 0.08	60a ± 4	1.15ab ± 0.15	1.19 ± 0.32	1.6a ± 0.2	138ab ± 14
Juice from M	31c ± 3	15.2e ± 1.2	nd c	12.5d ± 2.8	nd e	2.86d ± 0.08	36b ± 3	0.88d ± 0.07	nd	nd	98e ± 9
Juice from MHW	43d ± 3	7.3f ± 0.1	6.0a ± 0.8	18.5ac ± 3.6	1.6b ± 0.0	1.17ab ± 0.08	51c ± 3	1.31bc ± 0.09	nd	1.4a ± 0.2	131a ± 9
Juice from MSB	63a ± 1	3.6g ± 0.2	6.5a ± 1.7	22.7ab ± 1.3	1.3ab ± 0.0	1.61b ± 0.15	63a ± 1	1.38c ± 0.06	nd	3.3b ± 0.1	166b ± 4

A—glucoraphanin, B-glucothiobeinin, C-glucosativin, D-glucoerucin, E-unknown, F-glucobrassicin, G-7-dimeric 4-mercaptobutyl-GLS, H-4-metoxyglucobrassicin, I-gluconasturtiin, J-unknown

*M* mash from leaves without additional pre-treatment, *MHW* mash from leaves after heated treatment in water, *MSB* mash from leaves after steam blanching

Means within columns marked by the same letter do not differ significantly at *P *< 0.05, nd—not detected

Pretreatment of comminuted leaves with hot water and steaming resulted in a 10% and 34% reduction in contents of GLS respectively, in comparison to the raw material. Both pretreatment with hot water and steaming leaves contributed to a significant decrease (*P* < 0.05) in the contents of glucoraphanin, glucosativin, glucothiobeinin. However, in the case of steaming the reduction in the contents of these glucosinolates was significantly higher (*P* < 0.05). Concentration of glucobrassicin, dimeric 4-mercaptobutyl and 4-methoxyglucobrassicin increased significantly (*P* < 0.05) as a result of pretreatment with hot water. This may have been connected with the greater recovery factors of these compounds from tissue. Gliszczyńska-Świgło et al. (2006) reported a 1.1-fold and 1.4–1.6-fold increase of the content of aliphatic and indole glucosinolates in broccoli upon thermal pretreatment in comparison to fresh broccoli. A 35% increase in total extractable glucosinolates after microwave treatment of red cabbage in comparison to the raw material was found by Oerlemans et al. (2006).

In the control (M) and comminuted leaves after steaming (MSB), glucobrassicin, dimeric 4-mercaptobutyl and 4-methoxyglucobrassicin contents were comparable to those in the raw material (Table 1). There are different studies that indicate an advantageous effect of steaming limiting losses of glucosinolates in cruciferous vegetables. Volden et al. (2008b) when investigating the effect of water blanching, boiling and steam blanching of red cabbage chunks; recorded 64%, 38% and 19% reduction of glucosinolate contents, respectively. Boiling, water blanching and steam blanching applied for various cauliflower varieties caused losses of GLS amounting to 55%, 42% and 19%, respectively (Volden et al. 2009). Francisco et al. (2010) stated that boiling of turnip greens and turnip tops contributed to glucosinolate losses amounting to 64%, while after steam blanching the losses ranges from 9 to 21%. However, losses caused by water blanching to a considerable degree are reported as dependent on the amount of the solution and may range from 18 to 82% (Dekker et al. 2000; Sones et al. 1984). Normally in this study, tested samples were treated with a 10% of water (relatively smaller), thus higher retention of these amounts may have depended on the water amount. Moreover, the effect of thermal treatment on glucosinolate content in the raw material is dependent on the type of raw material.

Glucosinolate concentrations of the raw juice from steamed leaves, hot water treatment and no pretreatment were 79%, 62% and 47% of total content, respectively (calculated on the basis of values in Table 1). The greater contents of these compounds in raw juices obtained from thermally treated leaves may be connected both with the higher pressing efficiency and inactivation of myrosinase. There are not many studies describing the content of glucosinolates in juices. Hallmann et al. (2017) showed that glucosinolates were detectable in fresh cabbage but not in sauerkraut juice. These authors suggest, it was connected with glucosinolates decomposition during fermentation process and may be related to myrosinase activity.

In the juice produced from the leaves which were treated with hot water and steam, the concentrations of glucoraphanin, dimeric 4-mercaptobutyl, glucosativin, glucoerucin, 4-metoxyglucobrassicin were found higher in comparison to raw juice produced from untreated mash. In the case of glucothioibeinin and glucobrassicin the highest content, among the tested juices, was found in the raw juice produced from untreated leaves (Table 1).

### Content of phenolic compounds

In rocket leaves, quercetin derivatives accounting for 55% of total phenolic compound content and the rest composed of hydroxycinnamic acids (Table 2). Taranto et al. (2016) when analysing wild rocket salads from various accessions also found quercetin derivatives as a dominant among flavonoids. They recorded flavonoid contents at comparable levels of 42–73 mg/100 g f.w. (assuming dry weight at 8.5% based on our determinations). Available literature data indicate a higher share (80–90%) of flavonoids in the total content of phenolic compounds (Heimler et al. 2007; Taranto et al. 2016). However, these differences may be connected with growing conditions. For instance, Selma et al. (2010) reported the effect of soil amendments prepared from organic wastes (sewage sludge and urban solid waste) on a reduction in the content of flavonols in rocket leaves (*Eruca sativa*).

**Table 2:** Phenol compounds content (mg/100 g f.w.) in the mash and the juices from wild rocket leaves (n = 9, mean ± standard deviation)

Sample	Hydroxycinamic acids	Derivatives quercetin	Total of phenolic compounds content
Raw material	38.5e ± 4.9e	47.8f ± 1.3	86.3b ± 3.6
M (control)	35.8e ± 0.8e	47.2f ± 0.2	83.0b ± 1.0
MHW	31.1c ± 0.7c	52.0g ± 2.3g	83.1b ± 3.0
MSB	19.7d ± 0.8	38.0d ± 0.4	57.7d ± 0.5
Juice from M	12.3a ± 0.1a	21.3a ± 0.7a	33.6a ± 0.8
Juice from MHW	16.1b ± 1.9	29.3c ± 0.9c	45.4c ± 2.9
Juice from MSB	13.2ab ± 0.2a	24.b 1 ± 0.2	37.3a ± 0.2

*M* mash from leaves without additional pre-treatment, *MHW* mash from leaves after heated treatment in water, *MSB* mash from leaves after steam blanching

Means within columns marked by the same letter do not differ significantly at *P* < 0.05

In mash obtained from hot water pretreatment leaves and no pretreatment (control), the content of phenolic compounds was the same as in the raw material. In samples from steamed leaves, their contents were 33% lower. Steaming reducing specific phenolic content, hydroxycinnamic acids by 49% and quercetin derivatives by 21% compare to the raw material. For quercetin derivatives the highest recovery rates were observed in samples obtained from leaves pretreated with hot water, even more phenolic compounds passed compared to the raw material (Table 2). Some authors suggested, that better recovery rates of phenolic compounds can be reached with the thermal treatment (Puupponen-Pimia et al. 2003). Most literature data indicate greater retention of phenolic compounds in Brassica vegetables as a result of steam blanching rather than water blanching (Zhang and Hamauzu 2004; Pellegrini et al. 2010). For example, Volden et al. (2008a) when investigating the effect of various thermal treatment methods for red cabbage found a 43% decrease in phenolic contents in water blanched samples, in which the water: raw material ratio was 1:10. In the case of steam blanching they reported increased contents of analysed compounds. In our study, the highest recovery rates of phenolic compounds were observed in mash from hot water pretreatment leaves. However, this thermal treatment was applied with a 10% water usage, which may have limited losses of the analysed compounds. The effect of this factor was indicated in a study by Zhang and Hamauzu (2004), who found as much as a 72% reduction in phenolic compounds in broccoli florets boiled for 5 min using a vegetable–water ratio of 1:20. Vegetable variety was considered as an important factor for this result. This was indicated by a study that Amin and Lee (2005) conducted with water blanching application, who recorded a decrease in phenolic contents for red cabbage, white cabbage, mustard cabbage and Chinese white cabbage, while the content of phenolics increased in Chinese cabbage at the application of identical water blanching conditions.

The highest content of phenolic compounds among the tested juices was recorded in raw juice produced from hot water treatment leaves (53% of total of the phenolic compound content in raw material). In raw juices from untreated leaves (control) or steamed leaves the content of phenolic compounds was approx. 20% lower. Hallmann et al. (2017) recorded, that the flavonoid content in sauerkraut juice was 50% lower than in raw material. The level of hydroxycinnamic acids in wild rocket raw juices ranged from 12.3 to 16.1 mg/100 g. In the case of quercetin derivatives the highest content was found in raw juices produced from leaves subjected to pretreatment with hot water, followed by those from steamed leaves and untreated leaves (control) (Table 2). A lower content of phenolic compounds in raw juices produced from untreated leaves (control) or steamed leaves may be the result of either a lack or incomplete inactivation of polyphenol oxidase. Wang et al. (2019) also reported that thermal processed kale juices exhibited higher total phenolics than the cold-pressed juice. These authors indicate that the thermal processing might have removed sugar moiety from flavonoid glycosides in kale to get free hydroxyl groups. Therefore, thermal-processed juices had higher phenolic compound content and radical scavenging activities compared to cold-pressed juices.

### Antioxidant capacity

Antioxidant capacity recorded for the raw material and for the mash ranged from 6.7 to 10.4 μmol/g f.w. Steaming pretreatment caused to significant decrease in antioxidant capacity (*P* < 0.05). In raw juices the highest antioxidant capacity was found for samples obtained from mash from leaves subjected to pretreatment with hot water (Table 3). A strong correlation (r = 0.93) was observed between contents of phenolic compounds and antioxidant capacity. A significant correlation between antioxidant capacity and phenolic content for leaves of various Brassicaceae species (including salad rocket) (r = 0.86) was also reported by Martínez-Sánchez et al. (2008). Heimler et al. (2007) also indicated such kind of correlation in other raw materials from Brassicaceae family (broccoli, cabbage) with the exception of cauliflower.

**Table 3:** Antioxidant capacity (μmol Trolox/1 g f.w.) in the mash and the juices from wild rocket leaves (n = 9, mean ± standard deviation)

Sample	ABTS
Raw material	9.1de ± 0.3
M	10.4e ± 0.7
MHW	7.6cd ± 1.5
MSB	6.7bc ± 1.4
Juice from M	3.3a ± 0.1
Juice from MHW	5.8b ± 0.0
Juice from MSB	3.9a ± 0.3

*M* mash from leaves without additional pre-treatment, *MHW* mash from leaves after heated treatment in water, *MSB* mash from leaves after steam blanching

Means within columns marked by the same letter do not differ significantly at *P* < 0.05

### Cytotoxic activity of wild rocket raw juice

**Fig. 1:**
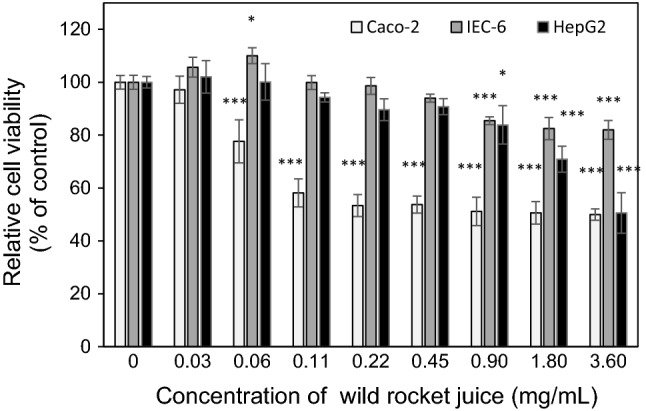
The effect of wild rocket juice on the cell viability and metabolic activity measured using the MTT test. Statistically significant differences *P < 0.05, **P < 0.01, ***P < 0.001 versus control group

Wild rocket raw juice obtained from pretreatment with hot water leaves was evaluated for its cytotoxic activity to the cells originating from the gastrointestinal tract, including the small intestine (IEC-6 cell line), colon (Caco-2 cell line) and the liver (HepG2 cell line). As shown in Fig. 1, the impact of wild rocket raw juice on cell proliferation and viability is strictly dose-dependent. The raw juice at the lowest concentration (0.03 mg/ml) did not affect the cell culture growth (*P* > 0.05). The initial cytotoxic activity at which wild rocket raw juice reduced the metabolic activity of the IEC-6, Caco-2 and HepG2 cells by 10% (EC_10_), were calculated at 0.66 ± 0.03 mg/mL, 0.04 ± 0.01 mg/mL and 0.46 ± 0.02 mg/mL, respectively (Table 4). Increasing the concentration of wild rocket raw juice in Caco-2 and HepG2 cell cultures caused a significant limitation of cell growth and decrease of cell viability (Fig. 1), which was reflected by the value of median effective concentration (EC_50_). The wild rocket raw juice concentrations responsible for a 50% reduction in Caco-2 and HepG2 cell viability were estimated at 1.87 ± 0.08 mg/mL and 3.54 ± 0.29 mg/mL, respectively (Table 4). As shown in Fig. 1, the raw juice at the maximum concentration (3.6 mg/mL) reduced viability and metabolic activity of the IEC-6 cells by only 19.04 ± 1.2%. The results indicate that wild rocket raw juice exerts the strongest cytotoxic activity on colon Caco-2 cells (*F *= 54.0; *P *< 0.0001), followed by HepG2 hepatocytes (*F *= 31.2; *P *< 0.0001), and it shows the lowest cytotoxic activity in the culture of IEC-6 cells (*F *= 27.5; *P *< 0.0001) from the small intestinal epithelium (Fig. 1).

**Table 4:** Cytotoxic doses of wild racket juice determined in IEC-6, Caco-2 and HepG2 cell cultures (n = 3, mean ± standard deviation)

Cytotoxic dose	IEC-6 cells	Caco-2 cells	HepG2 cells
EC_10_ (mg/mL)	0.66 ± 0.03	0.04 ± 0.01	0.46 ± 0.02
EC_50_ (mg/mL)	> 3.60/nd	1.87 ± 0.08	3.54 ± 0.29

nd—non-detected in the concentration range from 0.03 to 3.60 mg/mL

The cytotoxic activity of wild rocket on cancer cells was poorly investigated. Durazzo et al. (2013) describes such impact of polyphenolic wild rocket extract on Caco-2 cells for doses of 0.05 mL/mL and 0.10 mL/mL. Cytotoxic activity of wild rocket raw juice may be related to the content of both phenolic compounds and glucosinolates. Therefore, further studies should be targeted to define the mechanisms of cytotoxic activity.

## Conclusion

Conducted study show that hot water pretreatment of leaves and steaming caused a 10% and 34% reduction in contents of GLS in comparison to the raw material, respectively. In the produced raw juices the highest glucosinolate contents were recorded for samples from steamed leaves, followed by those from hot water treated leaves and untreated leaves (control). The greater contents of these compounds in raw juices obtained from thermally treated leaves may be connected both with the higher pressing efficiency and inactivation of myrosinase. In mash obtained from hot water pretreatment leaves and no pretreatment (control), the content of phenolic compounds was the same as in the raw material. In samples from steamed leaves, their contents were 33% lower. The highest content of phenolic compounds was found in raw juices produced from leaves pretreated with hot water. This may have resulted from differences in the distribution or binding of these compounds with the tissue of the tested raw material. Studies have also shown that wild racket juice in physiologically relevant doses does not induce cytotoxic effects on small intestinal epithelial cells. In contrast, treatment of colorectal cancer cells with low doses of wild rocket juice resulted in a significant reduction in cell proliferation and viability, which may suggest its anticancer potential. However, these preliminary in vitro studies should be continued in the future with a detailed analysis of anti-proliferative mechanisms, including cell cycle progression and induction of apoptosis.
